# Molecular Programming of Drought-Challenged *Trichoderma harzianum*-Bioprimed Rice (*Oryza sativa* L.)

**DOI:** 10.3389/fmicb.2021.655165

**Published:** 2021-04-13

**Authors:** Bishnu Maya Bashyal, Pooja Parmar, Najam Waris Zaidi, Rashmi Aggarwal

**Affiliations:** ^1^Division of Plant Pathology, ICAR-Indian Agricultural Research Institute, Pusa, New Delhi, India; ^2^International Rice Research Institute, Pusa, New Delhi, India

**Keywords:** *Trichoderma harzianum*, drought, rice, transcriptome (RNA-seq), DEGs

## Abstract

*Trichoderma* biopriming enhances rice growth in drought-stressed soils by triggering various plant metabolic pathways related to antioxidative defense, secondary metabolites, and hormonal upregulation. In the present study, transcriptomic analysis of rice cultivar IR64 bioprimed with *Trichoderma harzianum* under drought stress was carried out in comparison with drought-stressed samples using next-generation sequencing techniques. Out of the 2,506 significant (*p* < 0.05) differentially expressed genes (DEGs), 337 (15%) were exclusively expressed in drought-stressed plants, 382 (15%) were expressed in *T. harzianum*-treated drought-stressed plants, and 1,787 (70%) were commonly expressed. Furthermore, comparative analysis of upregulated and downregulated genes under stressed conditions showed that 1,053 genes (42%) were upregulated and 733 genes (29%) were downregulated in *T. harzianum*-treated drought-stressed rice plants. The genes exclusively expressed in *T. harzianum-*treated drought-stressed plants were mostly photosynthetic and antioxidative such as plastocyanin, small chain of Rubisco, PSI subunit Q, PSII subunit PSBY, osmoproteins, proline-rich protein, aquaporins, stress-enhanced proteins, and chaperonins. The Kyoto Encyclopedia of Genes and Genomes (KEGG) enrichment analysis states that the most enriched pathways were metabolic (38%) followed by pathways involved in the synthesis of secondary metabolites (25%), carbon metabolism (6%), phenyl propanoid (7%), and glutathione metabolism (3%). Some of the genes were selected for validation using real-time PCR which showed consistent expression as RNA-Seq data. Furthermore, to establish host–*T. harzianum* interaction, transcriptome analysis of *Trichoderma* was also carried out. The Gene Ontology (GO) analysis of *T. harzianum* transcriptome suggested that the annotated genes are functionally related to carbohydrate binding module, glycoside hydrolase, GMC oxidoreductase, and trehalase and were mainly upregulated, playing an important role in establishing the mycelia colonization of rice roots and its growth. Overall, it can be concluded that *T. harzianum* biopriming delays drought stress in rice cultivars by a multitude of molecular programming.

## Introduction

Rice is an important staple crop, feeding more than half of the world’s population. Due to the high demand of water for maintenance of its growth and productivity, the yield of the crop is severely hampered by the limited availability of water.

Drought is one of the major constraints that affect crop productivity up to 9–10% globally due to its adverse effects on plant growth and development (Lesk et al., 2016). Hence, to combat drought stress and bring an environment-friendly solution to the problem, biological management practices have also been explored. Among fungi, *Trichoderma harzianum* association is also known for its positive effects in combating abiotic and biotic stresses (Contreras-Cornejo et al., 2013). *T. harzianum* is an antagonistic mycoparasite that colonizes the roots of both monocots and dicots (Schuster and Schmoll, 2010). Additionally, *T. harzianum* biopriming also leads to enhanced nutrient uptake and increased water-holding capacity (Doni et al., 2014), which ultimately results in better growth and development in drought-stressed soils (Bae et al., 2009). The *Trichoderma* association helps plants in sustaining drought stress by increasing the expression of antioxidative enzymes, secondary metabolites, and plant hormones (Pandey et al., 2016; Alwhibi et al., 2017). Bashyal et al. (2020) also reported that seed priming with *T. harzianum* delayed drought stress by 3–5 days.

*Trichoderma*-mediated improved growth is also due to alterations in plant physiological parameters related to anabolic pathways such as photosynthetic rate, stomatal conductance, and transpiration rate (Doni et al., 2014). The root colonization of *Trichoderma* is often associated with enhanced photosynthesis as evident through various studies (Doni et al., 2014; Harman et al., 2019). Previous studies suggested that *Trichoderma* colonization improves the plant biomass and root growth by increasing auxin biosynthesis (Contreras-Cornejo et al., 2009). A few transcriptomic studies have also been carried out to correlate the genomic basis of *T. harzianum*-induced physiological and biochemical changes in drought tolerance (Huang et al., 2014; Doni et al., 2019). A microarray study showed that genes related to oxidative and osmotic stress are induced upon *Trichoderma* root colonization to eliminate salinity stress (Brotman et al., 2013). Transcriptomic studies to understand molecular reprogramming in tomato plants treated with *T. harzianum* revealed that epigenetic responses and alternative splicing play a crucial role in plant growth and defense. Furthermore, a temporal regulation of ethylene/indole acetic acid and reactive oxygen species-mediated upregulation of defense mechanisms were also reported for the maintenance of growth and stress tolerance (Palma et al., 2019).

Previous transcriptome conducted by Ereful et al. (2020) on rice cultivars suggested the upregulation of genes of secondary metabolites interacting with reactive oxygen species and enhancement of antioxidative genes. However, there is a complete lack of study related to transcriptome profiling in the association of *T. harzianum* with rice cultivars in drought stress modulation. Hence, in the present study, we compared transcriptomic alterations in drought-stressed *T. harzianum-*inoculated rice plants using next-generation sequencing techniques. The results of this study may contribute to elucidating the mechanisms involved in the rice–microbe–drought interactions and to identify genes that are putatively responsible for the *T. harzianum*-mediated drought tolerance.

## Materials and Methods

### Seed Material and Source

Two contrasting rice cultivars were used in this experiment. A drought-resistant (Sahbhagi Dhan) and a drought-susceptible (IR64) genotype of rice and a biocontrol agent *T. harzianum* 1 (TH1) isolate were provided by the International Rice Research Institute, New Delhi, India. The TH1 strain was selected on the basis of our previous study (Bashyal et al., 2020).

### Inoculation and Plant Growth Conditions

The seeds were sown after surface sterilization for 1 min with 1% (v/v) sodium hypochlorite followed by washing three times with sterilized distilled water. The seeds were then bioprimed with powder formulation of *T. harzianum* at a concentration of 10 g/kg supplied with 0.02% (v/v) Tween 20 as surfactant. The formulation was mixed thoroughly to provide uniform coating and kept in a moist chamber at room temperature (25°C) for 24 h. The control surface-sterilized seeds were mixed with Tween 20 [0.02% (v/v) Tween 20] only and kept in the moist chamber. The seeds were then planted in earthen pots containing sterilized sand:soil (3:1) mixture and grown under day/night temperatures of 30–35°C/18°C and a relative humidity of 80/90%. After 40 days of plant growth, the potted plants were categorized into three groups: one group was used as control with normal irrigation (Sahbhagi Dhan, IR64), the second was drought-stressed (Sahbhagi Dhan drought-stressed and IR64 drought-stressed), and the third was drought- and biocontrol-treated (Sahbhagi Dhan, *T. harzianum-*treated + drought-stressed and IR64, *T. harzianum-*treated + drought-stressed). The drought stress was given as described previously by Bashyal et al. (2020). Briefly, moisture was maintained in the potted plants by applying 100 ml of water per pot every alternate day until plants attained the age of 40 days, and at this point, drought treatments were given by altering the water cycle. Watering was stopped for the subsequent days for each drought treatment (4, 7, and 10 days drought stress), while the control seedlings were watered every alternate day. The experiment was conducted in randomized block design with three replications per treatment and 10 seeds per pot. Overall, 90 plants for each cultivar (30 controls, 30 drought-stressed, and 30 *T. harzianum-*treated + drought-stressed) were observed for one time interval (i.e., 4 days drought stress). The plant samples were harvested after 4, 7, and 10 days of drought treatment and stored immediately using liquid nitrogen at −80°C for transcriptome analysis (Bashyal et al., 2016).

### Total RNA Extraction

Total RNA was isolated from the frozen plant samples (IR64, drought-stressed; IR64, *T. harzianum*-treated + drought-stressed) using TRIzol (TRI reagent, Molecular Research Center, OH, United States) following the manufacturer’s instructions. RNA isolation was done in two replicates from the pooled samples (4, 7, and 10 days). Briefly, a 100-mg seedling of rice was powdered using liquid nitrogen, homogenized in TRIzol, and incubated for 5 min at room temperature. Then, 200 μl of chloroform was added to it and incubated for 10 min at room temperature after shaking vigorously. Samples were centrifuged (Eppendorf AG, Heidelberg, Germany) at 12,000 rpm for 15 min and the upper aqueous phase was separated. Five hundred microliters of isopropanol was added to it and incubated at room temperature for 5 min. RNA was pelleted by centrifuging at 10,000 rpm for 10 min and purified by washing twice with 75–80% (v/v) alcohol. The RNA was dissolved in 40–50 μl of nuclease-free water and kept on a water bath set at 55–60°C. The quality and quantity of the isolated RNAs were checked on denatured RNA agarose gel and NanoDrop (Thermo Fisher Scientific, Wilmington, DE, United States) reading, respectively.

### RNA-Seq Library Construction and Sequencing

RNA-Seq paired-end sequencing libraries were prepared from the isolated total RNA using Illumina TruSeq stranded mRNA sample preparation kit (Illumina, San Diego, CA, United States). For this, mRNA was enriched from the total RNA using poly-T attached magnetic beads, followed by enzymatic fragmentation and first strand cDNA was synthesized. The first strand cDNA was then synthesized to second strand using second strand mix and Act-D mix to facilitate RNA-dependent synthesis. The double strand cDNA samples were then purified using Ampure XP beads (New England Biolabs, Ipswich, MA, United States) followed by A-tailing, adapter ligation, and then enriched by a limited number of PCR cycles.

### Transcriptome Sequencing, Quality Control, and Mapping

Sequencing was done in a single HiSeq 4000 lane using 150 bp paired-end chemistry. The library preparation and sequencing was done by commercial service providers (NxGenBio Life Sciences, New Delhi, India). The barcoded gDNA libraries were pooled in equal ratios and used for 2 × 150-bp paired-end sequencing on a single lane of the Illumina HiSeq 4000. Illumina clusters were generated and were loaded onto Illumina Flow Cell on Illumina HiSeq 4000 instrument and sequencing was carried out using 2 × 150-bp paired-end chemistry. After sequencing, the samples were demultiplexed and the indexed adapter sequences were trimmed using the CASAVA v1.8.2 software (Illumina Inc.).

### Data Analysis

#### Bioinformatics Analysis

The quality of raw reads was checked by FastQC (version 0.11.8). The high-quality reads were mapped using Minimap (version 2.17) at default parameters against both the reference genome *Oryza sativa* (NCBI acc. no. PRJNA13141) and *T. harzianum* 1 (NCBI acc. nos. PRJNA453596, PRJNA207867).

#### Differential Expression Analysis

The number of reads mapped to genes was calculated using SAMtools (version: 0.1.19). Differential analysis of all possible combinations was done using DESeq (version 1) R package, used to analyze count data from high-throughput sequencing assays such as RNA-Seq and test for differential expression. The functional annotation was done using UniProt and the Kyoto Encyclopedia of Genes and Genomes (KEGG) database. Expression plots (volcano plot) were made using In House R scripts and heat map using the MeV software.

#### Gene Expression Analysis by Quantitative Real-Time PCR

Quantitative real-time reverse transcriptase PCR (qRT-PCR) was done to validate the results of the Illumina sequencing experiment. For this, some of the significant candidate genes (Supplementary Table 1) contributing to drought tolerance and exclusively expressed in *T. harzianum*-treated and drought-stressed rice plants were selected and primers were designed (PrimerQuest tool, Integrated DNA Technologies). All the qRT-PCR experiments were conducted in three biological replicates with three technical replicates. For gene expression analysis, first RNA was extracted as stated above. Then, 3 μg of total RNA was used for cDNA synthesis using Verso cDNA synthesis kit (Thermo Scientific, Wilmington, DE, United States) according to the manufacturer’s protocol. For this, 3–5 μg of total RNA was taken in a microfuge tube. To this, nuclease-free water was added to make up the volume to 9 μl followed by the addition of different reagents in an indicated order as follows: 2 μl random hexamer, 1 μl of an RT enhancer, 4 μl of a 5× cDNA buffer, 2 μl of a 10-mM dNTP mix, and 1 μl of M-MuLV reverse transcriptase; then, it was mixed gently and spun slowly at 1,000 rpm for 15 s. The tubes were incubated at 40°C for 60 min. The reaction was terminated by heating at 70°C for 15 min.

The PCR reaction mix was prepared using primer pairs specific to rice (Supplementary Table 1), and GAPDH was used as internal control (Kumar et al., 2018). The reaction comprises 1 μl of cDNA mixed with 10 μl of SYBR Green PCR master mix (Qiagen GmbH, Hilden, Germany), 5 pmol of a forward primer, and 5 pmol of a reverse primer in a final volume of 20 μl. Template controls were analyzed for all genes. PCR was performed using a MiniOpticon real-time PCR system (Bio-Rad, Hercules, CA, United States) with the following conditions: an initial activation step at 94°C for 4 min, denaturation at 94°C for 15 s, annealing at 58°C for 30 s, and extension at 70°C for 30 s. Melt curve analysis of the PCR product was carried out at 72°C for 1 min and ramped from 75 to 90°C with an increase by 1°C every 5 s. The specificity of the reaction was confirmed by melt curve analysis and gel electrophoresis. Relative gene expressions were calculated in terms of fold changes using the ΔCt method [Fold change = 2^–Δ(ΔCt)^, ΔCt_*treated*_ = Ct(target) − Ct(normalizer), ΔCt_*control*_ = Ct(target) − Ct(normalizer), Δ(ΔCt) = ΔCt(treated) – ΔCt(control)]. The results are presented as arithmetic means and standard deviations of the replicates.

## Results

### Plant Growth

The comparative results of the two cultivars (Sahbhagi Dhan and IR64) showed that *T. harzianum* treatment has a significant difference in maintaining plant growth under drought conditions. The cultivar IR64 performed better when compared with Sahbhagi Dhan which was evident from plant growth which was assessed by measuring root and shoot length at 4, 7, and 10 days (Supplementary Figure 1). The root growth showed marginal difference on 4 and 7 days of growth; however, a notable difference was observed in shoot growth in *T. harzianum*-treated drought-stressed IR64 rice plants when compared with drought-stressed IR64. Sahbhagi Dhan recorded a 23% decrease in shoot length, whereas in IR64, a 6% decrease was recorded on 4 days of drought when compared with their respective controls. In fact, on 7 days of drought stress, Sahbhagi Dhan recorded a 60% less growth, whereas IR64 recorded 11% less growth in shoot length in comparison with their controls. Our previous study also indicated that *Trichoderma* biopriming delays drought stress by 3–5 days in IR64 and Sahbhagi Dhan (Bashyal et al., 2020). Overall, out of the two contrasting cultivars, i.e., Sahbhagi Dhan and IR64, the *T. harzianum-*inoculated IR64 cultivar performed better under drought stress at 4–7 days of growth (Supplementary Figure 2). Hence, cultivar IR64 was selected for the transcriptome analysis under *T. harzianum*-treated drought stress condition.

### Sequencing Statistics

The libraries of a total of four samples (IR64 drought; IR64 drought + *T. harzianum* in duplicates) were analyzed from Illumina NextSeq 500 platform. Approximately 20 million for drought-stressed samples and 21–22 million high-quality reads for *T. harzianum-*treated drought-stressed rice cultivar were obtained (Table 1). The alignment results showed that 86–95% of clean reads were mapped on the reference genome from all the four samples. The assembly of mapped reads resulted in the identification of a total of 33,691 differentially expressed genes (DEGs).

**TABLE 1:** Statistics of transcriptome sequencing results.

Sample	Mapping percent on reference genome			
IR64 *T. harzianum* + drought A	95.88			
IR64 *T. harzianum* + drought B	92.24			
IR64 drought A	89.28			
IR64 drought B	86.41			
Differential statistics when the reference was *Oryza sativa*				

**Sample**	**Total DEGs**	**DEGs at log_2_ FC**	**DEGs at log_2_ FC and *p* < 0.05**	**Number of annotated genes (at log_2_ FC and *p* < 0.05)**

IR64 *T. harzianum* + drought vs drought	33,691 ± 366	10,435 ± 2,098	2,506 ± 224	2,413 ± 224
*T. harzianum*	24,452 ± 1,664	11,884 ± 2,863	808 ± 76	806 ± 77

### Gene Profile and Differential Expression Study of Rice

**FIGURE 1:**
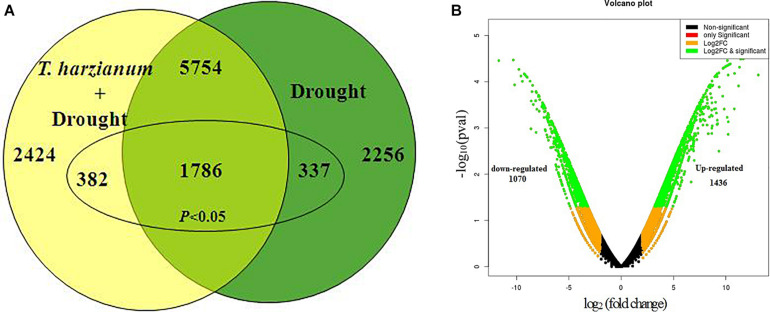
**(A)** Venn diagram showing the unique (non-overlapping region) and common expressed genes (overlapping region) obtained when drought-stressed rice is compared with *T. harzianum-*treated + drought-stressed rice. **(B)** Volcano plot representing significant and non-significant differentially expressed gene (DEG)-based *p* values. The green dot represents the significant DEGs.

Out of the total of 33,691 DEGs, the highly upregulated and downregulated 10,435 DEGs were considered for further studies excluding the genes lying in the range of log fold change in (−2) to (+2). Volcano plot analysis of DEGs showed remarkable differences in gene distribution patterns, which were aptly delimited in the *T. harzianum*-treated drought-stressed IR64 cultivar when compared with drought-stressed (Figure 1A). A total of 2,424 genes, i.e., 23%, were exclusively expressed in *T. harzianum-*treated plants, whereas 2,256 (22%) were expressed in drought-stressed conditions and 5,754 (55%) genes were commonly expressed in both treatments (Figure 1B). The study further stressed on 2,506 significant genes (*p* < 0.05) out of the highly expressed 10,435 genes; 337 (15%) were exclusively expressed in drought-stressed plants, 382 (15%) were expressed in *T. harzianum*-treated drought-stressed plants, and 1,787 (70%) were commonly expressed in both. Furthermore, comparative analysis of upregulated and downregulated genes under stressed conditions was also observed in *T. harzianum*+ drought vs drought-treated rice. The data showed 1,436 genes (57%) were upregulated and 1,070 genes (42%) were downregulated in *T. harzianum*-treated drought-stressed rice plants when compared with only drought-stressed plants.

**FIGURE 2:**
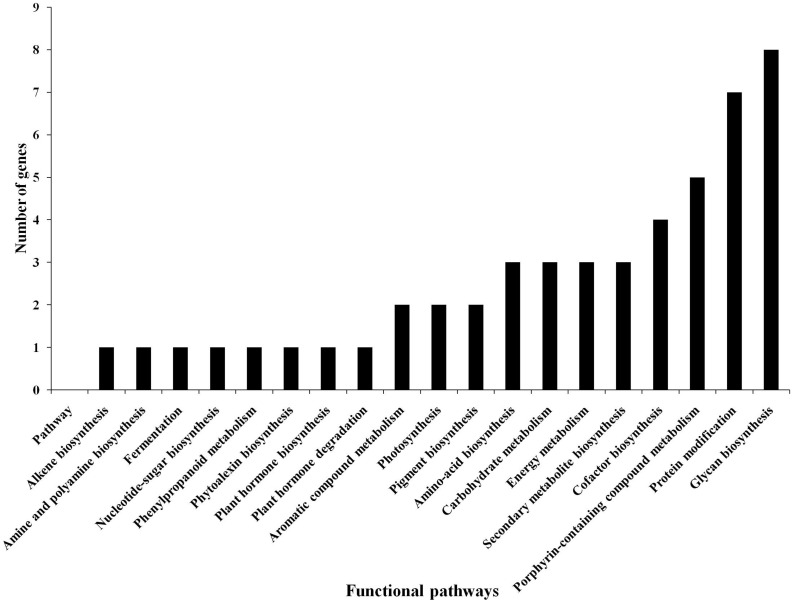
Commonly expressed functional pathway categories in the *T. harzianum*-treated drought-stressed rice genome vs drought-stressed rice.

**FIGURE 3:**
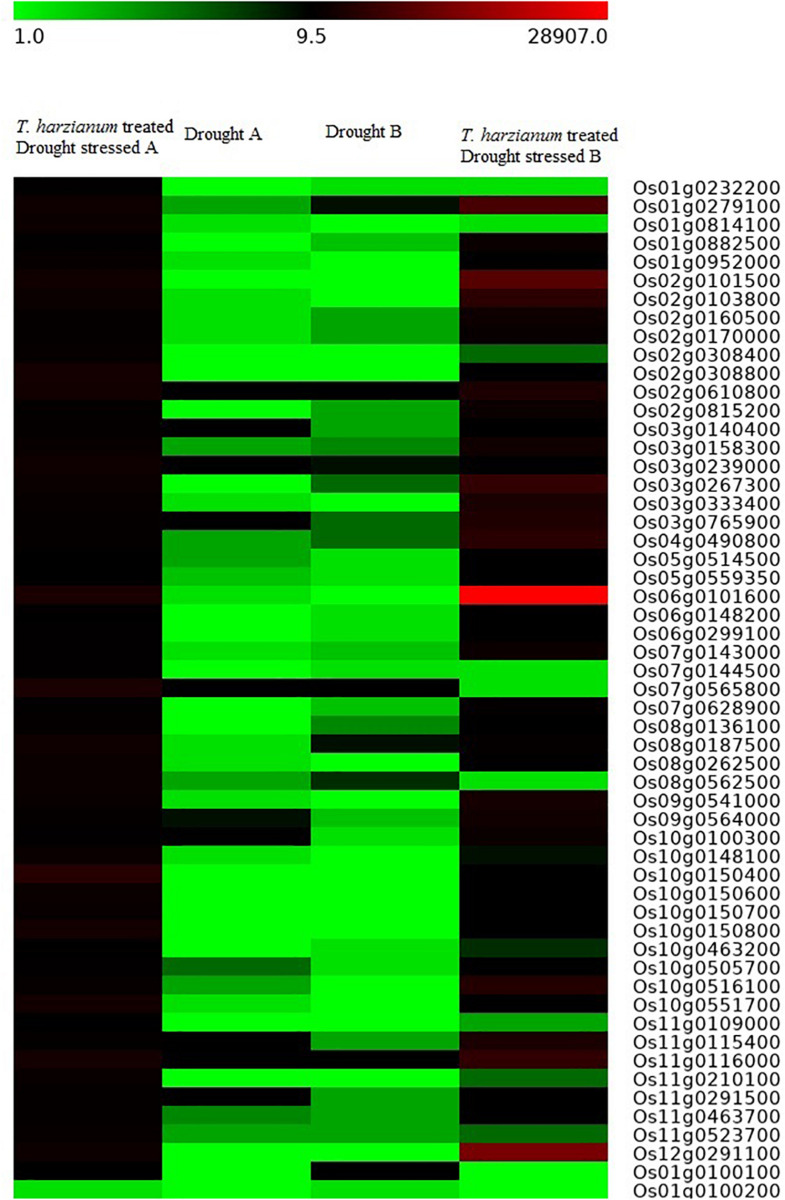
Heat map with cluster categorization representing the top 50 significant DEGs at two different comparisons of treatments (*T. harzianum*-treated + drought-stressed, drought-stressed). Each column represents the DEGs in different samples with two replicates. The red color shows upregulated genes and the green color represents downregulated genes based on highest FPKM values. Each row represents an individual transcript.

Based on the transcriptome analysis of DEGs under drought-stressed and *T. harzianum-*bioprimed conditions, the genes were classified into 21 broad categories (Figure 2). Heat map and hierarchical cluster categorization of DEGs were also generated to represent the global view of gene expression patterns and also depict their dynamic differences in *T. harzianum*-treated, drought-stressed vs drought-stressed (Figure 3). Higher percentages of genes were related to photosynthetic enzymes of both light reaction and dark reaction enzymes and osmotic homeostasis enzymes (Figure 3). The genes exclusively expressed in *T. harzianum*-treated plants were mostly photosynthetic such as plastocyanin, small chain of Rubisco, PSI subunit Q, and PSII subunit PSBY. Other antioxidative genes included osmoproteins, proline-rich protein, aquaporins, stress-enhanced proteins, chaperonins, peroxidases, and peroxiredoxins.

### Gene Profile and Differential Expression Study of *Trichoderma harzianum*

To establish rice–*T. harzianum* interaction, transcriptome analysis of *T. harzianum* was also carried out. The number of sequences obtained was 532 for a total length of 40,980,648 bp. The N50 length was 2,414,909 and the maximum and minimum contig lengths were 4,089,932 and 1,013 bp, respectively (Table 1). It has been generally accepted that larger values of performance criteria are associated with better assembly performance. An account of the DEGs suggests that there were 808 (3%) significant genes out the total DEGs (Table 1). GO analysis suggested that the annotated genes were functionally related to carbohydrate binding module (GO:0019867; GO:0016021), glycoside hydrolase (GO:0004553; GO:0005975), GMC oxidoreductase (GO:0016614; GO:0050660), and trehalase (GO:0005737). All the genes activated were involved in establishing the mycelia colonization of root and root growth.

### Gene Ontology Study

**FIGURE 4:**
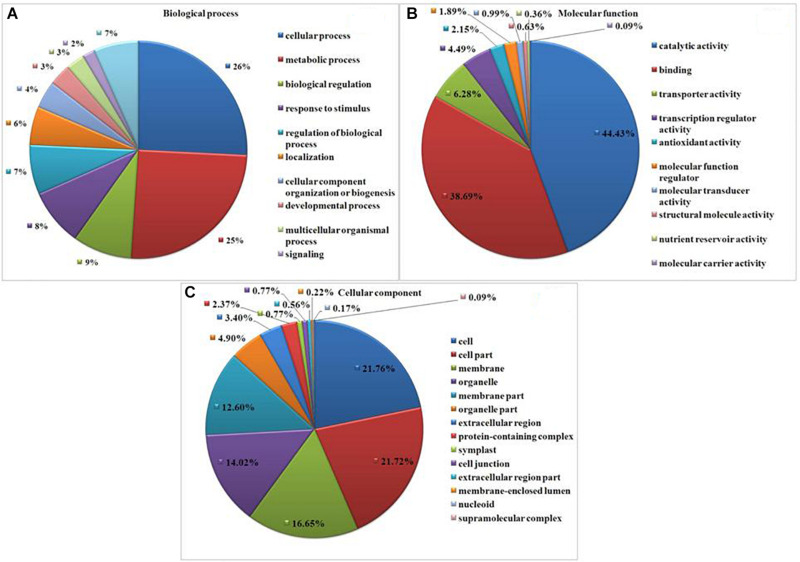
Gene Ontology (GO)-based functional annotation of genes present in the *T. harzianum*-treated drought-stressed rice genome vs. drought-stressed rice. **(A)** Biological process domains, **(B)** molecular function domain, and **(C)** cellular process domains.

Gene Ontology (GO) analysis revealed GO representation in drought and *T. harzianum*-primed drought samples. Among the significantly expressed DEGs, 1,657 (66%) GO terms were assigned for *T. harzianum*-treated drought-stressed vs drought-stressed samples. Cellular processes (39.5%, GO:0009987) and metabolic process (38.5%, GO:0008152) were the most significantly represented groups in the biological process category. Within the cellular component category, cell (50.6%, GO:0005623) and cell part (50.5%; GO:0044464) were the most significantly represented groups, and catalytic activity (49.5%, GO:0003824) was the most significantly represented group within the molecular function category (Figure 4). Supplementary Table 3 provides the list of highly upregulated significant genes expressed in *T*. *harzianum*-treated cultivars.

### KEGG Pathway and Enrichment Analysis

**FIGURE 5:**
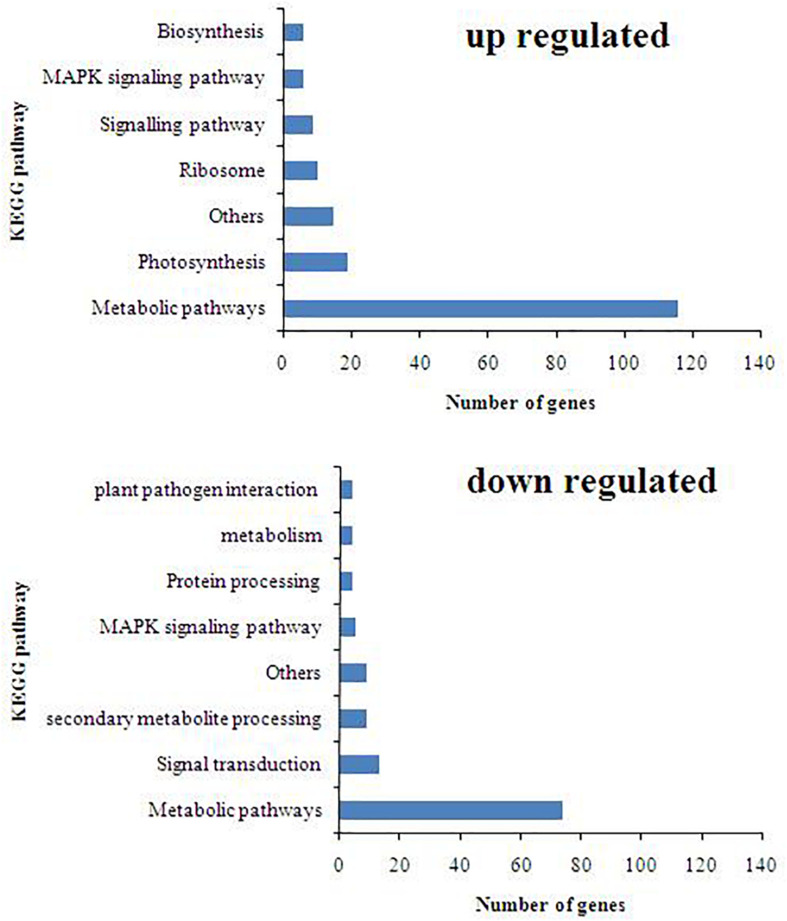
KEGG pathway distribution of upregulated and downregulated genes.

The KEGG pathway database was used to identify the role of *T. harzianum*-induced genetic pathway to reduce drought stress in rice plants. It was found that upon *T. harzianum* priming, a number of genes were upregulated and downregulated in drought-stressed rice plants (Figure 5). The exclusively expressed genes in *T. harzianum-*inoculated metabolic pathway were mostly related to glutathione metabolism (GO:0004364; GO:0005737; GO:0005576; GO:0006979), steroid biosynthesis (GO:0000254; GO:0005506; GO:0005789), and carbon metabolism (GO:0005739; GO:005960; GO:0019464). The highly upregulated genes included were related to photosynthesis (GO:0009416; GO:0009522; GO:0009523; GO:0009535).

**FIGURE 6:**
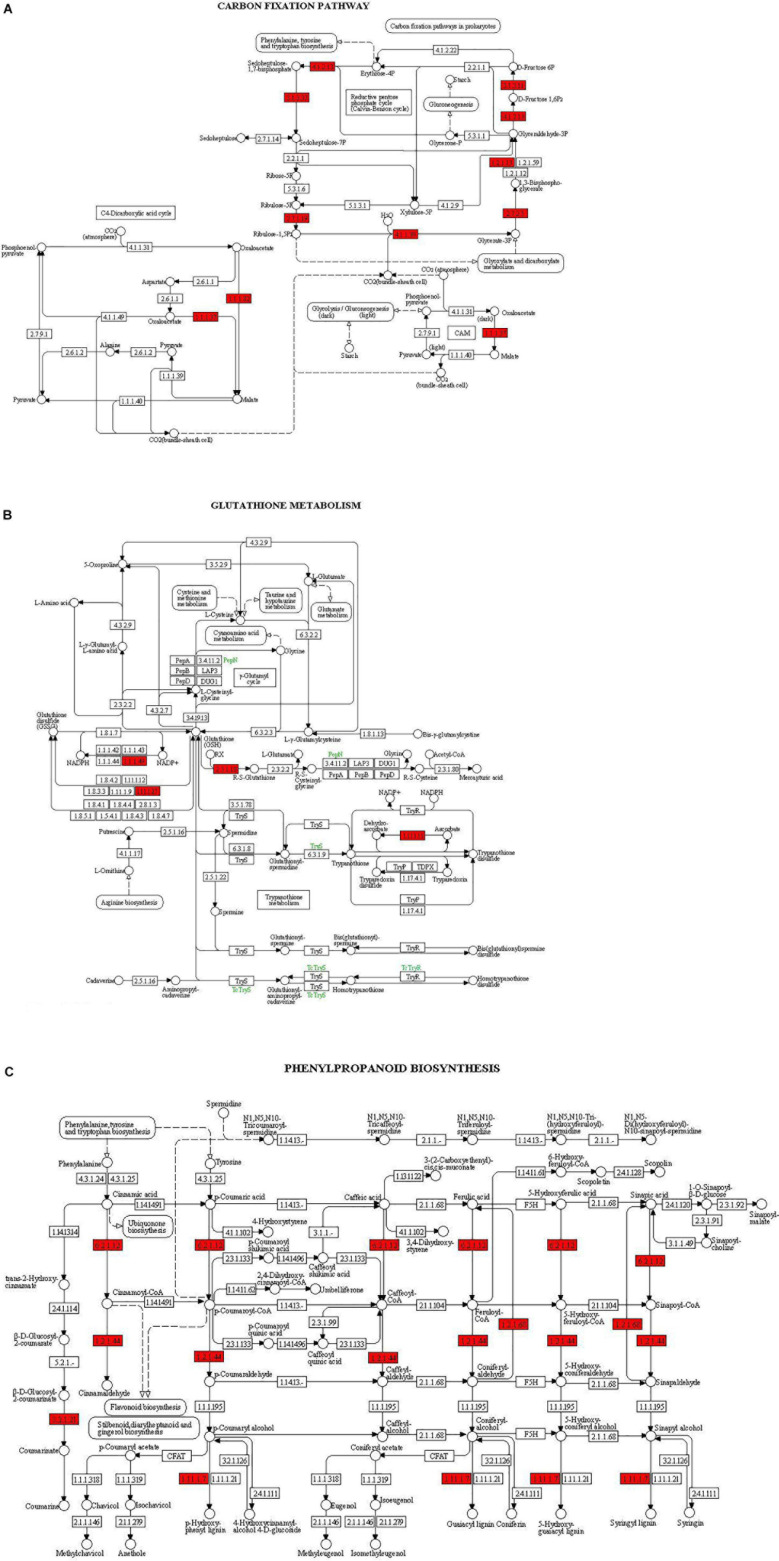
KEGG enrichment for DEGs from the three pathways: **(A)** carbon fixation, **(B)** glutathione metabolism, and **(C)** phenyl propanoid biosynthesis. The red highlights represent the enriched enzymes of the pathways.

The main pathways enriched in *T. harzianum*-treated rice relative to drought stress were related to metabolic pathways involved in the synthesis of secondary metabolites followed by pathways related to “photosynthesis.” The proteins of the photosystems and the enzymes of the Calvin cycle were upregulated. Overall, it can be concluded that *T. harzianum* helped rice plants by increasing the expression of photosynthetic genes, phenyl propanoid pathway genes, glutathione pathway, stress-enhanced osmoproteins, etc. (Figures 6A–C).

To further investigate the DEGs that were involved and enriched in various metabolic pathways, pathway-based analysis was performed using the KEGG pathway database. In this, a total of 468 significant (*p* < 0.05) genes of the KEGG pathway were found to be enriched. The genes were classified into 13 different categories (Supplementary Table 2). Broadly, the most enriched pathways were metabolic (38%) followed by pathways involved in the synthesis of secondary metabolites (25%), carbon metabolism (6%), phenyl propanoid (7%), and glutathione metabolism (3%).

### Validation of Differentially Expressed Genes Through Real-Time PCR

**FIGURE 7:**
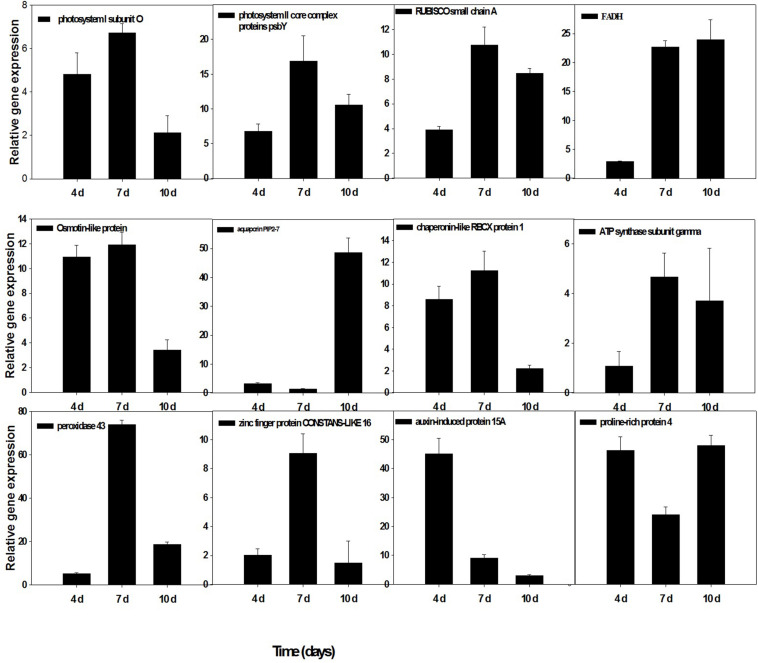
qRT-PCR validation of selected genes showed significant difference in their expression in *T. harzianum*-treated and drought-stressed when compared with drought-stressed at three different time intervals (4, 7, and 10 days). Error bars show ± SD among the biological replicates.

To confirm the accuracy and reproducibility of the Illumina RNA-Seq results, 12 representative genes were chosen to validate the levels of expression in drought and *T. harzianum*-primed drought-stressed IR64 cultivar by qRT-PCR. The validation results for the selected genes are shown in Figure 7. Out of the 12 genes, five genes—ribulose-bisphosphate carboxylase small chain A, ferredoxin–NADP reductase, chaperonin-like RBCX protein 1, ATP synthase subunit gamma, and photosystem II core complex proteins psbY (Os12g0291100, Os02g0103800, Os08g0425200, Os07g0513000, and Os08g0119800)—were chloroplastic. Three genes such as proline-rich protein (Os10g0150400), osmotin-like protein (Os12g0569500), and aquaporin PIP2 (Os09g0541000) were related to the maintenance of osmotic homeostasis of rice plants. Besides these, PSI subunit (Os04g0414700), peroxidase 43 (Os11g0210100), stress-enhanced protein 1 (Os11g0621400), and auxin-induced protein 15A (Os08g0118500) expression were also assessed. The expressions of all the genes were consistent with RNA-sequencing data.

## Discussion

*Trichoderma harzianum* is an antagonistic biocontrol agent used widely as a plant growth promoter, in alleviation of abiotic stresses such as drought, salinity, and heat stress (Rawat et al., 2012; Ahmad et al., 2015; Angel Contreras-Cornejo et al., 2016). In fact, seed biopriming has shown promising results in decreasing the effect of drought stress as evident from the increase in growth, photosynthetic parameters such as net photosynthetic rate, stomatal conductance, increase in the activity of antioxidative enzymes, secondary metabolites, and related decrease in peroxides (Rawat et al., 2016).

First, our results compared the plant growth of two contrasting cultivars, i.e., IR64 and Sahbhagi Dhan. IR64 was selected for further study due to its better performance. *T. harzianum-*mediated better growth has been reported in various plants such as rice, tomato, and maize (Morán-Diez et al., 2015; Bashyal et al., 2020). The increase in growth is attributed to the decrease in drought stress by improvement in water use efficiency, osmotic balance by increased proline content, and decrease in MDA content (Pandey et al., 2016; Mishra et al., 2020). The molecular analysis has shown that plant growth promotion might be due to an enhanced expression of carbohydrate binding module family protein (M431DRAFT_521249, M431DRAFT_499139), glycoside hydrolase (M431DRAFT_95191), and polysaccharide lyase (M431DRAFT_92754) as observed in our results. The growth promotion due to *T. harzianum* colonization may in part be due to cellulose disruption by induction of Tg SWO, carbohydrate binding module, and swollenin protein (Brotman et al., 2008; Meng et al., 2019). The carbohydrate binding module-mediated root elongation is mediated by cellulose breakdown, which results in cellular expansion thereby resulting in cellular signaling for cell growth (Brotman et al., 2008).

Our study showed that *T. harzianum* biopriming has proved beneficial in withstanding drought stress. The stress tolerance might be correlated to the upregulation of various genes which have been identified to be involved in carbon metabolism and carbon fixation. Among DEGs, some genes which were exclusively expressed or are highly upregulated with respect to drought stress play a significant role in drought stress alleviation. The log fold changes of the exclusively expressed genes such as Os06g0101600 (plastocyanin, chloroplastic-like), Os12g0291100 (ribulose-bisphosphate carboxylase small chain A), Os02g0103800 (ferredoxin–NADP reductase, leaf isozyme 2, chloroplastic-like), Os08g0425200 (chaperonin-like RBCX protein 1, chloroplastic), and Os07g0513000 (ATP synthase subunit gamma, chloroplastic) were infinite as the reads were recorded for *T. harzianum*-treated rice only and not for drought-treated cultivars (Supplementary Table 3). The log fold changes for highly upregulated genes such as Os04g0414700 (photosystem I subunit O), Os08g0119800 (photosystem II core complex proteins psbY, chloroplastic), Os12g0569500 (osmotin-like protein), Os02g0815200 (28 kDa ribonucleoprotein, chloroplastic), Os04g0490800 (phosphoglycolate phosphatase 1B, chloroplastic), and Os01g0882500 [NAD(P)H-quinone oxidoreductase subunit N, chloroplastic] were 10.2, 10, 9.7, 7, 7.1, and 7.2, respectively (Supplementary Table 3). The increase in expression of photosynthetic genes is consistent with previous results (Azarmi et al., 2011; Alwhibi et al., 2017; Doni et al., 2019). The increase in photosynthetic genes might be due to an increase in phytohormones also observed in our results, for instance Os03g797800 (auxin-responsive protein) and Os01g0883800 (gibberellin oxidase) which are upregulated by 5.41 and 5.29 log fold change. Additionally, the carotenoids protect pigments from photooxidative stress (Strzaka et al., 2003) and our pathway analysis results indicated enriched enzymes of carotenoid biosynthesis.

The exclusive expression of peroxidase and the enrichment of glutathione metabolism pathway in *T. harzianum*-primed rice cultivar as observed in our study indicate toward the involvement of glutathione peroxidase pathway active in antioxidation ROS formed due to drought stress. The induction of antioxidant defense has also been observed in tomato seedlings and rice in water deficit after *T. harzianum* interaction (Mastouri et al., 2012; Singh et al., 2020). Similar results were observed when maize seedlings were inoculated with *Trichoderma atroviride* (Guler et al., 2016).

Besides, the genes involved in photosynthetic pathway, the secondary metabolite pathway genes such as phenylpropanoid, glutathione metabolism, diterpenoid, glyoxylate, nitrogen, cutin, and suberin were also upregulated in *T. harzianum*-inoculated drought-stressed IR64 rice cultivar. The antioxidative role of secondary metabolites and their effect in decreasing drought stress are well established (Tattini et al., 2014; Kubala et al., 2015). Glutathione is a tripeptide found abundantly in cellular components and gene functions in cell growth and regulation of stress-responsive genes (Sofo et al., 2015).

Additionally, we observed upregulation of osmotin-like proteins (Os12g38170) and aquaporins (Os0232900) in *T. harzianum*-primed drought-treated rice. Osmotin-like proteins are known to protect plants by maintaining cellular osmolarity by compartmentalization of solutes or by structural and metabolic alterations (Choi et al., 2013). In fact, their overexpression has been correlated with stress tolerance (Chowdhury et al., 2017; Bashir et al., 2020). The aquaporins are channel proteins which play a key role in plant responses to environmental stresses by maintaining the uptake and movement of water in the plant body and maintain cell viability (Kapilan et al., 2018). The upregulation is also linked to drought stress maintenance (Shekoofa and Sinclair, 2018).

Kyoto Encyclopedia of Genes and Genomes enrichment analysis was performed to identify related pathways for 468 genes that were involved and enriched in this study. The most enriched genes in carbon fixation pathway include malate dehydrogenase, sedoheptulose-bisphosphatase, ribulose-5-phosphate kinase, ribulose-bisphosphate carboxylase, fructose bisphosphate aldolase, glyceraldehyde-3-phosphate dehydrogenase, and phosphoglycerate kinase (Os02g0698000, Os03g0129300, Os03g0267300, Os04g0234600, Os04g0459500, Os05g0496200, Os06g0608700, Os07g0630800, Os08g0562100, Os11g0171300, and Os12g0274700). The genes enriched in glutathione pathway were glucose-6-phosphate dehydrogenase (Os07g0406300), glutathione-dependent peroxiredoxin (Os02g0192700), glutathione transferase (Os10g395400), and ascorbate peroxidase (Os02g2553200). The genes found to be enriched in phenyl propanoid pathway are coumarate-CoA ligase (Os02g0697400), cinnamoyl-coA reductase (Os09g0400000), coniferyl aldehyde dehydrogenase (Os04g0540600), beta glucosidase (Os09g0511900), and peroxidase (Os10g0109300).

Hence, overall, *T. harzianum* biopriming aided rice plants in a multifaceted simultaneous manner by triggering various pathways such as photosynthetic, secondary metabolites, and osmotic balance maintenance contributing to enhanced tolerance to drought stress (Singh et al., 2020).

## Conclusion

We provided a comprehensive study of the transcriptome of a drought-challenged IR64 rice cultivar bioprimed with *T. harzianum*. The complete analyses of DEGs are highlighted with respect to drought treatment. The data revealed that the *T. harzianum*-mediated drought stress improvement is associated with a synchronous induction of various metabolic pathways involved in photoprotection of photosynthetic apparatus, upregulation of photosynthetic genes, upregulation of antioxidative genes, ascorbate glutathione pathway, and maintenance of osmotic homeostasis by increasing proline, osmotic proteins, and aquaporins. Additionally, the transcriptome analysis of *T. harzianum* also supported the fact that its association helps in plant growth by the regulation of carbohydrate binding module polysaccharide lyase and glycoside hydrolase. Overall, it can be concluded that *T. harzianum* biopriming delays drought stress in rice cultivars by a myriad of molecular reprogramming.

## Data Availability Statement

The datasets generated for this study can be found in online repositories. The names of the 373 repositories and accession numbers can be found below: https://www.ncbi.nlm.nih.gov/, SRR12567752, SRR12567751, SRR12567750, and SRR12567749.

## Author Contributions

BB, NZ, and RA conceptualized the problem and designed the experiment. PP and BB conducted the experiment and analyzed the data. PP wrote the manuscript. BB, RA, and NZ edited the manuscript. All authors finalized the manuscript.

## Conflict of Interest

The authors declare that the research was conducted in the absence of any commercial or financial relationships that could be construed as a potential conflict of interest.

## Supplementary Material

Supplementary Figure 1 Effect of *T. harzianum* treatment in drought stressed rice cultivars’ (IR64 and Shahbhagi Dhan) root length **(A–C)** and shoot length **(D–F)** after different days (4, 7, and 10 days) of drought stress.

Supplementary Figure 2 Effect of *T. harzianum* treatment on plants of two rice varieties (V1, Shahbhagi Dhan and V2, IR64) after different days (4 and 7 days) of drought stress where a, b, and c represent control, *T. harzianum* treated + drought stress and drought stressed plants in each image.

Supplementary Table 1 List of primers used for expression analysis.

Supplementary Table 2 Enriched KEGG pathways of differentially expressed genes in *T. harzianum*-rice interaction.

Supplementary Table 3 List of differentially expressed upregulated genes.
