# Supplementary figures and images for: Molecular Programming of Drought-Challenged Trichoderma harzianum-Bioprimed Rice (Oryza sativa L.)
Source: Front Microbiol. 2021 Apr 13;12:655165. doi: 10.3389/fmicb.2021.655165 (PMC8076752; doi:10.3389/fmicb.2021.655165)

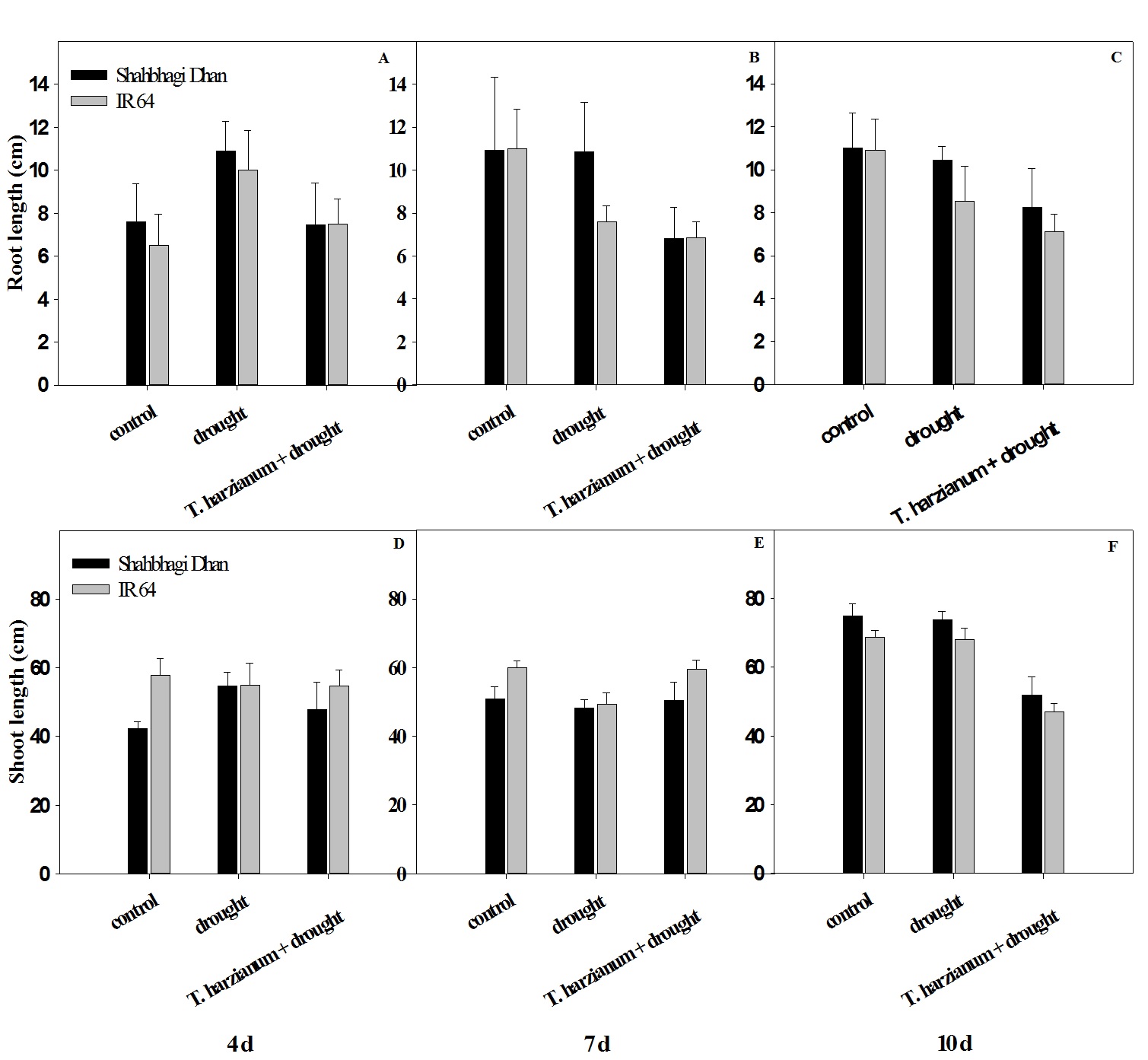

Supplement: Supplementary Figure 1 — Effect of T. harzianum treatment in drought stressed rice cultivars’ (IR64 and Shahbhagi Dhan) root length (A–C) and shoot length (D–F) after different days (4, 7, and 10 days) of drought stress. [file Image_1.jpeg]

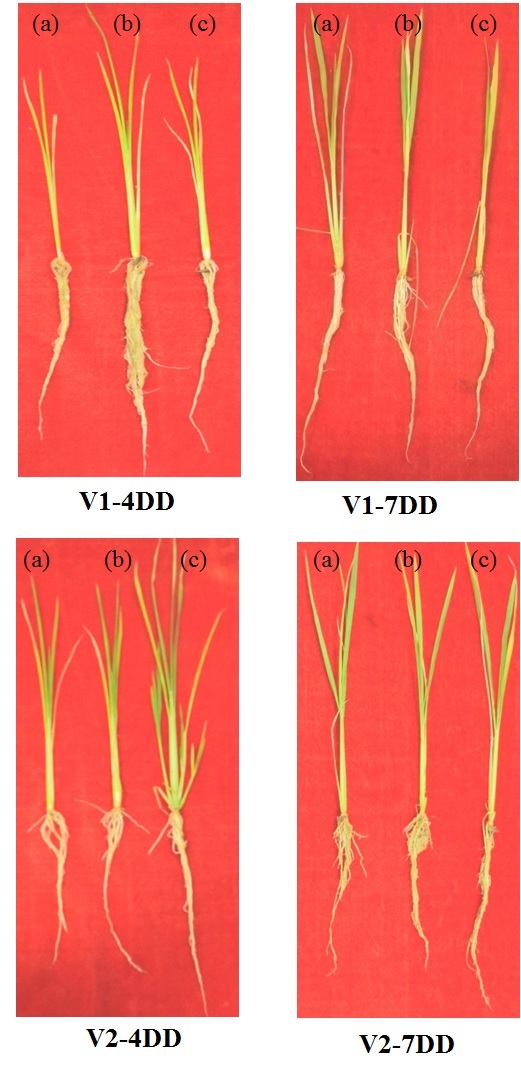

Supplement: Supplementary Figure 2 — Effect of T. harzianum treatment on plants of two rice varieties (V1, Shahbhagi Dhan and V2, IR64) after different days (4 and 7 days) of drought stress where a, b, and c represent control, T. harzianum treated + drought stress and drought stressed plants in each image. [file Image_2.jpeg]
