# Supplementary material for: Molecular Programming of Drought-Challenged Trichoderma harzianum-Bioprimed Rice (Oryza sativa L.)
Source: Front Microbiol. 2021 Apr 13;12:655165. doi: 10.3389/fmicb.2021.655165 (PMC8076752; doi:10.3389/fmicb.2021.655165)
Supplement: Supplementary Table 1 — List of primers used for expression analysis. [file Table_1.DOCX]

Supplementary table 1. List of primers used for expression analysis.

| **S.no.** | **Gene ID** | **Gene name** | **Primer sequence** |
| --- | --- | --- | --- |
| 1. | Os10g0150400 | proline-rich protein 4 | 5’TGCACCCGGAAGAACATGAA3’ 5’CCTGGAGGTCTGCACAAAAGA3’ |
| 2. | Os12g0291100 | ribulose bisphosphate carboxylase small chain A | 5’TACCTCCTCAGGCTTCTCCTTT3’  5’AAGGCACCCACTTGGATCG3’ |
| 3. | Os02g0103800 | ferredoxin--NADP reductase, leaf isozyme 2 | 5’CCGACTCCAAGACGGTAACT3’ 5’AGACTCCTAGCTTACAGAGGAA3’ |
| 4. | Os11g0210100 | peroxidase 43 | 5’TACTGCCTAGCTCGGTGACA3’  5’GATGAGGCTTACAGCTGAGGAG3’ |
| 5. | Os08g0425200 | chaperonin-like RBCX protein 1 | 5’GCTGTACAGGGAGGAGTTCG3’ 5’CTTGAGGCCGCACATGTAGA3’ |
| 6. | Os07g0513000 | ATP synthase subunit gamma | 5’AACCAGGAGATCCAGACGGA3’  5’GAAGTAGGCGTTGCCCTTCT3’ |
| 7. | Os11g0621400 | stress enhanced protein 1 | 5’CGAGCAAGAGGAGACCACTC3’ 5’GCTGCATTGATGCGCATAGG3’ |
| 8. | Os08g0118500 | auxin-induced protein 15A | 5’GAGGAATTCGGCTTCGACTG3’ 5’GAACACAAAAATCCGAGCCGT3’ |
| 9. | Os09g0541000 | probable aquaporin PIP2-7 | 5’ACCACGTGAGAGAGAACGAAC3’ 5’GTGCTCCTGAACGAGCTGAG3’ |
| 10. | Os04g0414700 | photosystem I subunit O | 5’TAGCAAAAGCGTTCAGTGGC3’ 5’GAAGGATATGCAGCGCACAC3’ |
| 11. | Os08g0119800 | photosystem II core complex proteins psbY | 5’TGAGCCGAAGCCAAAGTTCA3’ 5’AGCTAGCTAACCCAAACCCG3’ |
| 12. | Os12g0569500 | Oosmotin-like protein | 5’ATGGAATAACAAAGGCGGGGT3’ 5’TGGACGGATACATACTGTTGGA3’ |
