# Supplementary material for: Molecular Programming of Drought-Challenged Trichoderma harzianum-Bioprimed Rice (Oryza sativa L.)
Source: Front Microbiol. 2021 Apr 13;12:655165. doi: 10.3389/fmicb.2021.655165 (PMC8076752; doi:10.3389/fmicb.2021.655165)
Supplement: Supplementary Table 2 — Enriched KEGG pathways of differentially expressed genes in T. harzianum-rice interaction. [file Table_2.DOCX]

Supplementary table 2. Enriched KEGG pathways of differentially expressed genes in *T. harzianum*-rice interaction.

| **Functional Category** | **Enriched**  **Genes** | **Total genes** | **Percentage** |
| --- | --- | --- | --- |
| Metabolic pathways | 180 | 1601 | 38.46 |
| Biosynthesis of secondary metabolites | 117 | 840 | 25.00 |
| Carbon metabolism | 26 | 212 | 5.56 |
| Phenylpropanoid biosynthesis | 35 | 158 | 7.48 |
| Glutathione metabolism | 15 | 76 | 3.21 |
| Carbon fixation in photosynthetic organisms | 14 | 64 | 2.99 |
| Glyoxylate and dicarboxylate metabolism | 14 | 60 | 2.99 |
| Photosynthesis | 31 | 40 | 6.62 |
| Diterpenoid biosynthesis | 7 | 27 | 1.50 |
| Nitrogen metabolism | 6 | 23 | 1.28 |
| Carotenoid biosynthesis | 6 | 22 | 1.28 |
| Cutin, suberine and wax biosynthesis | 5 | 19 | 1.07 |
| Photosynthesis | 12 | 15 | 2.56 |
