# Supplementary material for: Molecular Programming of Drought-Challenged Trichoderma harzianum-Bioprimed Rice (Oryza sativa L.)
Source: Front Microbiol. 2021 Apr 13;12:655165. doi: 10.3389/fmicb.2021.655165 (PMC8076752; doi:10.3389/fmicb.2021.655165)
Supplement: Supplementary Table 3 — List of differentially expressed upregulated genes. [file Table_3.docx]

Supplementary table 3. List of differentially expressed up-regulated genes.

| **S no** | **Gene IDs** | **Protein name** | **log2 Fold Change** | **Gene ontology** | **Function** | **References** |
| --- | --- | --- | --- | --- | --- | --- |
| 1. | Os06g0101600 | Plastocyanin, chloroplastic-like | Infinite | Chloroplast thylakoid membrane [go:0009535]; copper ion binding [go:0005507]; electron transfer activity [go:0009055] | Act as mobile electron carrier between the Photosystem I reaction center complex and the cytochrome *b* _6_/*f* | Katoh (2003) |
| 2. | Os12g0291100 | Ribulose bisphosphate carboxylase small chain A, chloroplastic-like | Infinite* | Chloroplast [GO:0009507]; monooxygenase activity [GO:0004497]; ribulose-bisphosphate carboxylase activity [GO:0016984]; photorespiration [GO:0009853]; reductive pentose-phosphate cycle [GO:0019253] | Up regulates the transcript levels of the large subunit | Suzuki and Makino (2011) |
| 3. | Os02g0103800 | Ferredoxin--NADP reductase, leaf isozyme 2, chloroplastic-like | Infinite* | Chloroplast stroma [GO:0009570]; chloroplast thylakoid membrane protein complex [GO:0098807]; ferredoxin-NADP+ reductase activity [GO:0004324]; photosynthesis [GO:0015979] | Catalyze the final step in the photosynthetic electron transport chain | [Zhang and Cramer (2004](javascript:;)) |
| 4. | Os02g0148000 | Chloroplast import apparatus | 11.41 | Nucleus [GO:0005634]; regulation of transcription, DNA-templated [GO:0006355] | Chloroplast import apparatus coordinately up-regulates protein import and synthesis in leaf chloroplasts | Sun et al. (2009) |
| 5. | Os01g0303000 | Calvin cycle protein CP12-1 | 11.66 | Negative regulation of reductive pentose-phosphate cycle [GO:0080153] chloroplast [GO:0009507] | CP12 is a small, redox-sensitive protein, which aids in formation of a complex between glyceraldehyde-3-phosphate dehydrogenase and phosphoribulokinase | López-Calcagno et al. (2017) |
| 6. | Os12g0189400 | Photosystem I reaction center subunit N | 11.59 | Chloroplast photosystem I [GO:0030093]; photosynthesis [GO:0015979] | PSI-N affects the oxidation of plastocyanin required for plant health | Haldrup et al. (1998, 1999) |
| 7. | Os06g0320500 | Chlorophyll a-b binding protein 1B-21 | 10.23 | Photosynthesis, light harvesting in photosystem I [GO:0009768]; protein-chromophore linkage [GO:0018298]; response to light stimulus [GO:0009416]; chloroplast thylakoid membrane [GO:0009535]; integral component of membrane [GO:0016021]; photosystem I [GO:0009522]; photosystem II [GO:0009523]; chlorophyll binding [GO:0016168] | LHCB are apoproteins of light harvesting complexes associated with chlorophyll and xanthophylls and serves as the antenna complex | [Jansson (1994](javascript:;)), (1999) |
| 8. | Os07g0148900 | Photosystem I subunit | 10.22 | \| Photosystem I [GO:0009522];  Photosynthesis [GO:0015979] \| \| --- \| | Binding of LHCII | Jensen et al. (2007) |
| 9. | Os04g0490800 | phosphoglycolate phosphatase 1B, chloroplastic | 7.14 | Phosphatase activity [GO:0016791]; dephosphorylation [GO:0016311] | Responsible for [catalyzing](https://en.wikipedia.org/wiki/Catalysis) the conversion of  [2-phosphoglycolate](https://en.wikipedia.org/wiki/2-Phosphoglycolate) into [glycolate](https://en.wikipedia.org/wiki/Glycolate) and [phosphate](https://en.wikipedia.org/wiki/Phosphate). Hence, plays a major role in photorespiratory 2-phosphoglycolate metabolism | Dellero et al. (2016) |
| 10. | Os06g0608700 | Fructose-bisphosphate aldolase | 6.62 | Cytosol [GO:0005829]; fructose-bisphosphate aldolase activity [GO:0004332]; fructose 1,6-bisphosphate metabolic process [GO:0030388]; glycolytic process [GO:0006096] | Plastids enhances growth and photosynthesis of plants | Uematsu et al. (2012) |
| 11. | Os06g0107700 | Ferredoxin--NADP reductase | 6.11 | Chloroplast stroma [GO:0009570]; chloroplast thylakoid membrane protein complex [GO:0098807]; ferredoxin-NADP+ reductase activity [GO:0004324]; photosynthesis [GO:0015979] | Involved in final step of linear electron flow transferring electrons from ferredoxin to NADP^+^ | Kozuleva et al. (2016) |
| 12. | Os01g0882500 | NAD(P)H- quinoneoxidoreductase subunit N, chloroplastic | 7.23 | Chloroplast envelope [GO:0009941]; chloroplast thylakoid membrane [GO:0009535]; oxidoreductase activity, acting on NAD(P)H, quinone or similar compound as acceptor [GO:0016655]; defense response to fungus, incompatible interaction [GO:0009817]; NADH dehydrogenase complex (plastoquinone) assembly [GO:0010258] | NAD(P)H-quinoneoxidoreductase or NAD(P)H dehydrogenase 1 subunit D shuttles electrons from NADH, via FMN and iron-sulphur (Fe-S) centers, to quinones in the respiratory chain | Melo et al. (2004) |
| 13. | Os10g0150400  Os04g0644400 | proline-rich protein 4 | Infinite,  13.11 | --** | Imparts tolerance to drought, salt and heat stress | Mellacheruvu et al. (2016); Gujjar et al. (2019) |
| 14. | Os12g0569500 | Osmotin like protein | 9.73 | Extracellular region [GO:0005576] | Osmotin like proteins maintains cellular osmolarity by compartmentalization of solutes or by structural and metabolic alterations | [Choi et al. (2013](https://www.frontiersin.org/articles/10.3389/fpls.2017.00410/full#B4)) |
| 15. | Os02g0232900 | Aquaporin NIP1-1 | 5.89 | Integral component of membrane [GO:0016021]; channel activity [GO:0015267] | Defense responses against biotic and abiotic stressors. | Afzal et al. (2016) |
| 16. | Os08g0425200 | Chaperonin-like RBCX protein 1, chloroplastic | Infinite* | Chloroplast thylakoid [GO:0009534]; protein folding chaperone [GO:0044183]; chaperone-mediated protein folding [GO:0061077]; response to cold [GO:0009409]; response to salt stress [GO:0009651]; response to water deprivation [GO:0009414] | Assist in protein folding of chloroplastic enzymes | Zhao and Liu (2018) |
| 17. | Os06g0264200 | zinc finger protein CONSTANS-LIKE 16 | 9.15 | Nucleus [GO:0005634]; zinc ion binding [GO:0008270]; regulation of transcription, DNA-templated [GO:0006355] | Induction of flowering | Lagercrantz and Axelsson (2000) |
| 18. | Os04g0396800 | Carboxypeptidase | 6.93 | Vacuole [GO:0005773]; serine-type carboxypeptidase activity [GO:0004185]; proteolysis involved in cellular protein catabolic process [GO:0051603] | Regulation of defense responses against biotic and oxidative stress | Liu et al. (2008) |
| 19. | Os11g0530600 | Chalcone synthase | 6.92 | Naringenin-chalcone synthase activity [GO:0016210]; flavonoid biosynthetic process [GO:0009813] | Its expression causes accumulation of flavonoid and isoflavonoid phytoalexins and is involved in the salicylic acid defense pathway. | Dao et al. (2011) |
| 20. | Os02g0744900 | Geranylgeranyl diphosphate reductase | 6.90 | Chlorophyll biosynthetic process [GO:0015995]; oxidation-reduction process [GO:0055114]; photosynthesis [GO:0015979]; vitamin E biosynthetic process [GO:0010189] | Geranylgeranyl diphosphate reductase maintains   tocopherol and phytylated Chl contents which otherwise negatively affects thylakoid membrane and affect photosynthetic machinery. | Tanaka et al. (1999) |
| 21. | Os08g0136100 | homeobox-leucine zipper protein ROC7 | 7.12 | Nucleus [GO:0005634]; lipid binding [GO:0008289]; sequence-specific DNA binding [GO:0043565]; regulation of transcription, DNA-templated [GO:0006355] | Members of HD-Zip class I are generally involved in abiotic stress responses such as water and light stress | Elhiti and Stasolla (2009) |
| 22. | Os04g0175900 | Probable inactive methyltransferase | 5.83 | O-methyltransferase activity [GO:0008171]; protein dimerization activity [GO:0046983]; S-adenosylmethionine-dependent methyltransferase activity [GO:0008757]; aromatic compound biosynthetic process [GO:0019438]; methylation [GO:0032259] | Plays a role in genome management and in regulating gene expression during development | Finnegan and Kovac (2000) |
| 23. | Os07g0628900 | cysteine-rich receptor-like protein kinase | 7.21 | Integral component of membrane [GO:0016021]; plasma membrane [GO:0005886]; ATP binding [GO:0005524]; protein serine/threonine kinase activity [GO:0004674]; protein phosphorylation [GO:0006468] | Disease resistance and cell death in plants | Quezada et al. (2019) |
| 24. | Os11g0243300 | Zinc-finger homeodomain protein | 6.96 | Nucleus [GO:0005634]; DNA binding [GO:0003677]; metal ion binding [GO:0046872] | Zinc-finger homeodomain protein gene family encodes a group of transcriptional regulators with unique biochemical activities that play overlapping regulatory roles in floral development. | Tan and Irish (2006) |
| 25. | Os10g0528900 | Glutathione S-transferase GSTU6 | 6.69 | Cytoplasm [GO:0005737]; glutathione transferase activity [GO:0004364]; glutathione metabolic process [GO:0006749] | Glutathione-*S*-transferases have been associated with detoxification of xenobiotics, limiting oxidative damage and other stress responses in plants. | Gong et al. (2005) |
| 26. | Os03g0832800 | Acyltransferase family protein | 6.63 | Integral component of membrane [GO:0016021]; membrane [GO:0016020]; glycerol-3-phosphate 2-O-acyltransferase activity [GO:0090447]; phosphatase activity [GO:0016791]; cutin biosynthetic process [GO:0010143] | Transfer an acyl moiety from energy-rich donor molecules to various acceptors, contributing to the vast diversification of plant secondary metabolites. | Ciarkowska et al. (2018) |
| 27. | Os09g0360500 | 3-ketoacyl-CoA synthase | 6.11 | Membrane [GO:0016020]; transferase activity, transferring acyl groups other than amino-acyl groups [GO:0016747]; fatty acid biosynthetic process [GO:0006633] | Catalyze first reaction in fatty acid elongation and involved in rice leaf cuticular wax synthesis | Wang et al. (2017) |
| 28. | Os11g0115400 | non-specific lipid- transfer protein 1-like | 7.1 | Membrane [GO:0016020]; lipid binding [GO:0008289]; lipid transport [GO:0006869] | They are involved in membrane stabilization cell wall organization and signal transduction | Liu et al. (2015) |
| 29. | Os06g0624700 | Glycosyltransferase | 6.55 | Transferase activity, transferring glycosyl groups [GO:0016757] | Glycosyltransferases can transfer sugar moieties from active sugar molecules to a variety of acceptor molecules, and are, hence, referred to as UGTs | Rehman et al. (2018) |
| 30. | Os06g0262800 | 3-ketoacyl-CoA synthase | 6.53 | Membrane [GO:0016020]; transferase activity, transferring acyl groups other than amino-acyl groups [GO:0016747]; fatty acid biosynthetic process [GO:0006633] | Ketoacyl synthases catalyze condensing reactions combining acyl-CoA or acyl-acyl carrier protein with malonyl-CoA to form 3-ketoacyl-CoA or with malonyl-ACP to form 3-ketoacyl-ACP thereby adding two carbon atoms to growing acyl chains | Chen et al. (2011) |
| 31. | Os11g0661600 | Peroxidase | 6.25 | Extracellular region [GO:0005576]; plant-type cell wall [GO:0009505]; plasmodesma [GO:0009506] hydrogen peroxide catabolic process [GO:0042744]; response to oxidative stress [GO:0006979] | Decompose hydrogen peroxide with the oxidation of phenolic and non- phenolic substrates | Pandey et al. (2017) |
| 32. | Os06g0196300 | Peroxiredoxin Q | 6.14 | Cell [GO:0005623]; chloroplast thylakoid lumen [GO:0009543]; chloroplast thylakoid membrane [GO:0009535]; cytoplasm [GO:0005737]; thioredoxin peroxidase activity [GO:0008379]; cell redox homeostasis [GO:0045454]; cellular response to oxidative stress [GO:0034599] | Peroxiredoxin Q functions as a monomeric protein and represents about 0.3% of chloroplast proteins. It attaches to the thylakoid membrane and is detected in preparations enriched in photosystem II complexes | Lamkemeyer et al. (2006); Dietz (2011) |
| 33. | Os09g0528000 | Kinesin-like protein KIN-7J | 6.06 | Kinesin complex [GO:0005871]; microtubule [GO:0005874]; ATP binding [GO:0005524]; ATPase activity [GO:0016887]; microtubule binding [GO:0008017]; microtubule motor activity [GO:0003777]; microtubule-based movement [GO:0007018] | Essential for oriented deposition of cellulose microfibrils and cell wall strength | Zhong et al. (2002) |
| 34. | Os02g0646500 | Aldehyde dehydrogenase | 5.98 | Integral component of membrane [GO:0016021]; 3-chloroallyl aldehyde dehydrogenase activity [GO:0004028]; cellular aldehyde metabolic process [GO:0006081] | Catalyze the oxidation of various aldehydes to the corresponding carboxylic acids, thus reducing the peroxidation of lipids | Sunkar et al. (2003) |
| 35. | Os02g0751600 | Peptidylprolyl isomerase | 5.97 | Chloroplast [GO:0009507]; chloroplast thylakoid membrane [GO:0009535]; peptidyl-prolyl cis-trans isomerase activity [GO:0003755] | They are responsible for cis/trans isomerization of the bond located before the proline residue and are involved in a variety of cellular processes, including protein folding. The role of immunophilins in thermoresistance control and saline as well as oxidative stresses have been observed. | Kaur et al. (2015) |
| 36. | Os03g0117100 | Peroxisomal membrane protein 11-1 | 6.24 | Integral component of peroxisomal membrane [GO:0005779]; peroxisomal membrane [GO:0005778] peroxisome fission [GO:0016559]; regulation of peroxisome size [GO:0044375] | Mediate various aspects of peroxisome biogenesis and maintenance, including the assembly of new membrane structures. | Ma et al. (2009) |
| 37. | Os02g0110200 | Hydroperoxide lyase | 5.91 | Green leaf volatile biosynthetic process [GO:0010597]; oxidation-reduction process [GO:0055114]; response to wounding [GO:0009611]; sterol metabolic process [GO:0016125] | Hydroperoxide lyases cleave the lipoxygenase products, resulting in the formation of omega-oxo acids and volatile C6- and C9-aldehydes and alcohols. It is known to be involved in wound healing and pest resistance | Noordermeer et al. (2001) |
| 38. | Os04g0175900 | Probable inactive methyltransferase | 5.82 | O-methyltransferase activity [GO:0008171]; protein dimerization activity [GO:0046983]; S-adenosylmethionine-dependent methyltransferase activity [GO:0008757]; aromatic compound biosynthetic process [GO:0019438]; methylation [GO:0032259] | Plays a role in genome management and in regulating gene expression during development. | Finnegan and Kovac (2000) |
| 39. | Os05g0458500 | Laccase-12 | 5.76 | Apoplast [GO:0048046]; plasma membrane [GO:0005886]; copper ion binding [GO:0005507]; ferroxidase activity [GO:0004322]; hydroquinone:oxygen oxidoreductase activity [GO:0052716]; iron ion homeostasis [GO:0055072]; iron ion transport [GO:0006826]; lignin catabolic process [GO:0046274] | Play roles in oxidizing monolignols to produce higher-order lignin involved in plant development and stress responses. | Liu et al. (2017) |
| 40. | Os07g0543100 | Beta-amylase | 5.56 | Amylopectin maltohydrolase activity [GO:0102229]; beta-amylase activity [GO:0016161]; polysaccharide catabolic process [GO:0000272] | The primary function of β-amylase is involvement in starch breakdown in plants. | Kossmann and Lloyd (2000) |
| 41. | Os10g0575600 | Homeobox-leucine zipper protein ROC3 | 5.55 | Regulation of transcription, DNA-templated [GO:0006355] nucleus [GO:0005634]; lipid binding [GO:0008289]; sequence-specific DNA binding [GO:0043565] | Members of HD-Zip class I are generally involved in abiotic stress responses such as water and light stress | Olsson et al. (2004) |
| 42. | Os02g0697400 | Probable 4-coumarate--CoA ligase 2 | 5.51 | Phenylpropanoid metabolic process [GO:0009698]; pollen exine formation [GO:0010584] 4-coumarate-coa ligase activity [GO:0016207]; ATP binding [GO:0005524] | CoA ligase gene (4CL) plays multiple important roles in plant growth and development by catalyzing the formation of CoA ester. 4CL belongs to the plant phenylpropane derivative, which is related to the synthesis of flavonoids and lignin and is a key enzyme in the biosynthetic pathway. | Chen et al. (2019) |
| 43. | Os03g0822000 | Expansin-A7 | 5.49 | Plant-type cell wall organization [GO:0009664] cell wall [GO:0005618]; extracellular region [GO:0005576]; membrane [GO:0016020] | Expansins are non-hydrolytic cell wall-loosening proteins that are involved in the cell wall modifications that underlie many plant developmental processes | Lin et al. (2011) |
| 44. | Os03g0797800 | Auxin-responsive protein | 5.41 | Auxin-activated signaling pathway [GO:0009734]; regulation of transcription, DNA-templated [GO:0006355] nucleus [GO:0005634] | Auxin-responsive GH3 gene family have been shown to play role in plant defense responses | Ghanashyam and Jain (2009) |
| 45. | Os01g0883800 | Gibberellin (GA) 20 oxidase 2 | 5.29 | Flower development [GO:0009908]; gibberellin biosynthetic process [GO:0009686]; response to flooding [GO:0009413]; response to light stimulus [GO:0009416]; unidimensional cell growth [GO:0009826] dioxygenase activity [GO:0051213]; gibberellin 20-oxidase activity [GO:0045544]; metal ion binding [GO:0046872] | GA20ox is a key enzyme that catalyzes the penultimate steps in GA biosynthesis. | Oikawa et al. (2004) |
| 46. | Os10g0100700 | Probable pyridoxal 5'-phosphate synthase subunit | 5.25 | Cellular amino acid metabolic process [GO:0006520]; pyridoxal phosphate biosynthetic process [GO:0042823]; pyridoxine biosynthetic process [GO:0008615] amine-lyase activity [GO:0016843]; pyridoxal 5'-phosphate synthase (glutamine hydrolysing) activity [GO:0036381] | It is an essential cofactor for numerous metabolic enzymes including amino acid metabolism and antibiotic biosynthesis | Tambasco-Studart et al. (2005) |
| 47. | Os03g0855100 | WRKY DNA binding domain containing | 5.20 | Nucleus [GO:0005634]; DNA-binding transcription factor activity [GO:0003700]; sequence-specific DNA binding [GO:0043565]; transcription regulatory region DNA binding [GO:0044212] | WRKY proteins play important role in the kinase signaling network. The interaction of plant resistance proteins with WRKY TFs and the existence of unusual chimeric R-WRKY proteins suggest diversity in signaling pathways for rapid immune responses. | Chen et al. (2019) |
| 48. | Os03g0637800 | Serine/threonine-protein kinase-like protein CR4 | 5.1 | Embryo development ending in seed dormancy [GO:0009793]; flower morphogenesis [GO:0048439]; lateral root formation [GO:0010311]; plant epidermal cell differentiation [GO:0090627]; plant organ development [GO:0099402]; protein autophosphorylation [GO:0046777]; regulation of asymmetric cell division [GO:0009786]; root cap development [GO:0048829]; Cell surface [GO:0009986]; endocytic vesicle [GO:0030139]; integral component of membrane [GO:0016021]; multivesicular body membrane [GO:0032585]; plasma membrane [GO:0005886]; ATP binding [GO:0005524]; protein homodimerization activity [GO:0042803]; protein kinase activity [GO:0004672]; protein serine/threonine kinase activity [GO:0004674] | Switching on or off the target proteins | Hardie (1999) |
| 49. | Os04g0494100 | Chitinase 5 | 5.10 | Chitin binding [GO:0008061]; chitinase activity [GO:0004568]; cell wall macromolecule catabolic process [GO:0016998]; chitin catabolic process [GO:0006032]; defense response [GO:0006952]; polysaccharide catabolic process [GO:0000272]; chitin binding [GO:0008061] | Chitinases play a dual role; they inhibit pathogenic fungal growth by cell wall digestion, and release pathogen-borne elicitors to induce defense reactions. | Sharma et al. (2011) |
| 50. | Os03g0683800 | GDSL esterase/lipase | 10.26 | Lipase activity [GO:0016298]; lipid metabolic process [GO:0006629] | GDSL esterase hydrolyzes thioesters, aryl esters, phospholipids and amino acids and plays significant roles in regulating plant growth and development. | [Akoh et al. (2004](https://www.frontiersin.org/articles/10.3389/fpls.2020.00726/full#B1)); [Ma et al. (2018](https://www.frontiersin.org/articles/10.3389/fpls.2020.00726/full#B37)) |

*Reads not present in drought stressed cultivar. **GO not defined
